# Workshop report: Toward the development of a human whole stool reference material for metabolomic and metagenomic gut microbiome measurements

**DOI:** 10.1007/s11306-020-01744-5

**Published:** 2020-11-08

**Authors:** Rupasri Mandal, Raul Cano, Cindy D. Davis, David Hayashi, Scott A. Jackson, Christina M. Jones, Johanna W. Lampe, Marie E. Latulippe, Nancy J. Lin, Katrice A. Lippa, Paulina Piotrowski, Sandra M. Da Silva, Kelly S. Swanson, David S. Wishart

**Affiliations:** 1 Department of Biological Sciences, University of Alberta, Edmonton, AB T6G 2E9 Canada; 2 The BioCollective, LLC, 5650 N Washington St, Denver, CO 80216 USA; 3 Office of Dietary Supplements, National Institutes of Health, Bethesda, MD 20852 USA; 4 Mondelez Global, LLC, Chicago, IL 60607 USA; 5 Material Measurement Laboratory, National Institute of Standards and Technology, Gaithersburg, MD 20899 USA; 6 Fred Hutchinson Cancer Research Center, 1100 Fairview Ave N, M4-B802, PO Box 19024, Seattle, WA 98109 USA; 7 North American Branch of the International Life Sciences Institute (ILSI North America), 740 15th Street NW, Suite 600, Washington, DC 20005 USA; 8 University of Illinois at Urbana-Champaign, Urbana, IL 61801 USA

**Keywords:** Metabolites, Metabolomics, Metagenomics, Nutrition, Diet

## Abstract

**Introduction:**

To date, there has been little effort to develop standards for metabolome-based gut microbiome measurements despite the significant efforts toward standard development for DNA-based microbiome measurements.

**Objectives:**

The National Institute of Standards and Technology (NIST), The BioCollective (TBC), and the North America Branch of the International Life Sciences Institute (ILSI North America) are collaborating to extend NIST’s efforts to develop a Human Whole Stool Reference Material for the purpose of method harmonization and eventual quality control.

**Methods:**

The reference material will be rationally designed for adequate quality assurance and quality control (QA/QC) for underlying measurements in the study of the impact of diet and nutrition on functional aspects of the host gut microbiome and relationships of those functions to health. To identify which metabolites deserve priority in their value assignment, NIST, TBC, and ILSI North America jointly conducted a workshop on September 12, 2019 at the NIST campus in Gaithersburg, Maryland. The objective of the workshop was to identify metabolites for which evidence indicates relevance to health and disease and to decide on the appropriate course of action to develop a fit-for-purpose reference material.

**Results:**

This document represents the consensus opinions of workshop participants and co-authors of this manuscript, and provides additional supporting information. In addition to developing general criteria for metabolite selection and a preliminary list of proposed metabolites, this paper describes some of the strengths and limitations of this initiative given the current state of microbiome research.

**Conclusions:**

Given the rapidly evolving nature of gut microbiome science and the current state of knowledge, an RM (as opposed to a CRM) measured for multiple metabolites is appropriate at this stage. As the science evolves, the RM can evolve to match the needs of the research community. Ultimately, the stool RM may exist in sequential versions. Beneficial to this evolution will be a clear line of communication between NIST and the stakeholder community to ensure alignment with current scientific understanding and community needs.

## Introduction and background

Over the past decade, it has become evident that the human gut microbiome and its metabolic by-products play an important role in a vast and disparate set of health and disease states, including inflammatory bowel disease, diabetes, obesity, cancer, and depression (Ahmed et al. 2013; Anand and Mande 2018; Wishart 2019a). A complex interrelationship of metabolic, hormonal, neurological, and immunological systems exists between the gut microbiome and the host. This molecular cross-talk is critical in regulating many physiological processes. Changes in the composition or function of the gut microbiome and its microbially derived metabolome can have profound consequences, both positive and negative, for the host (de Vos and de Vos 2012; Shreiner et al. 2015; Wishart 2019a).

### Challenges for gut microbiome and gut metabolome research

Traditionally, microbiome research focuses on the characterization and enumeration of microbial species using DNA analysis (Arndt et al. 2012; Knight et al. 2018). However, to truly understand the gut microbiome and the influence it has on human health, it is critical to look beyond identification of microbes. Among other important elements to examine is the chemistry that different gut microbes perform on our food as well as microbially produced metabolites more broadly. The human gut microbiome essentially functions as a metabolic organ that is vital to the digestion of food. Indeed, many of the chemical by-products arising from gut microbiome digestion play important roles in human metabolism, health, and disease. Short-chain fatty acids (SCFAs), such as butyric acid, are generated via the digestion of dietary fiber. These SCFAs have many positive health benefits, including anti-cancer and anti-inflammatory effects (Hoving et al. 2018; Primec et al. 2017). In contrast, many uremic toxins, such as indoxyl sulfate and hippuric acid, are produced by microbial biotransformation of dietary amino acids or dietary polyphenols (Shaw 2010; Tanaka et al. 2015; Wishart 2019a). Thus, the gut metabolome reflects the complex interaction of gut microbial activity and host genetics, diet, demographics, and health (Shah et al. 2020; Zierer et al. 2018).

A key challenge in both gut microbiome and gut metabolome research is collecting and analyzing samples that most accurately reflect microbial composition and metabolism and are of highest relevance to host health. It is well known that the microbial communities of fecal samples differ from those taken from the intestinal lumen or mucosa of healthy and diseased patients (Chen et al. 2014; Durbán et al. 2011; Rangel et al. 2015; Stanley et al. 2015). Because many of the fermentative metabolites are rapidly absorbed by the large intestinal mucosa, fecal samples are not a good measure of production and are a poor measure of microbial fermentative activity (Topping and Clifton 2001; Verbeke et al. 2015). The lack of intestinal sampling has hampered our ability to understand the influence of SCFAs on gut biological functions and host health (Dalile et al. 2019). This problem is likewise applicable to other metabolite classes produced by intestinal microbiota. Given the differences that exist among feces and the material present across the various intestinal regions, sampling the digesta or mucosa of the intestinal region of interest is preferred. However, although non-invasive collection devices may be available in the future (Hällgren 2003; Sinaiko 2004), feces are most commonly collected because invasive techniques (e.g., biopsies) are the only means by which intestinal samples may be collected currently and such sampling requires colon cleansing which can perturb the microbiome. Because of the invasiveness, these samples are usually limited to patients with disease or suspected disease so true controls are rarely available. Moreover, sample numbers are typically limited so studies require long periods of time.

Another key challenge is determining which gut microbial reactions are responsible for producing helpful or harmful metabolites and from which food products. Microbial metabolism is not only dependent on the foods that the host consumes, but also to what level the food components are digested and if/how gastrointestinal secretions (e.g., enzymes, mucus) have been affected. Because most of the microbial activity occurs in the large intestine, the metabolites produced by bacteria are primarily derived from the substances escaping host digestion in the stomach and small intestine. Currently, most investigations do not consider or estimate the host vs. microbial contribution to nutrient digestion, with very little being known about these aspects of gut microbiome metabolism. Using metabolomics, information has been compiled (Wishart 2019a; Wishart et al. 2018) about the bacteria that act on food components and which helpful/harmful chemicals are produced. This “catalogue,” however, is still very sparse. Furthermore, there has been very little action toward the development of standards for functional/metabolic measurements of gut microbiomes or gut-derived metabolomes.

### Value of a reference material for the field of fecal metabolomics

To understand biologically relevant properties of the human gut microbiome, validated analytical measurements that accurately describe various properties of the microbial community, both quantitatively and qualitatively, are needed. To date, DNA-based metagenomic measurements have been the go-to method for most microbiome studies (Knight et al. 2018; Riesenfeld et al. 2004). As a result, there have been significant efforts within the scientific community to increase confidence in metagenomic-based microbiome measurements. A few examples of these efforts include the development of reference materials (RMs) (mock communities and DNA mixtures), the launch of various interlaboratory comparison studies and measurement challenges to identify bias and assess reproducibility (e.g., https://platform.mosaicbiome.com/challenges/8), the organization of workshops and consortia that focus on standards development, and the development of better in vitro model systems that allow systematic studies across laboratories (Allen-Vercoe et al. 2019; Fritz et al. 2019; Hardwick et al. 2018; Li et al. 2019a; Sczyrba et al. 2017).

Despite the significant efforts devoted to the development of standards for DNA-based microbiome (especially gut microbiome) measurements, there has been little effort to develop standards for metabolome-based gut microbiome measurements. As a result, metabolomic data collected by different laboratories at different times cannot be readily compared, replicated, or studied in a systematic way. This has created a barrier to understanding links between the diet, the gut microbiome, and health (Karu et al. 2018).

With this need in mind, the National Institute of Standards and Technology (NIST), The BioCollective (TBC), and the North American Branch of the International Life Sciences Institute (ILSI North America) are collaborating to address the need for developing a Human Whole Stool RM. The intent of this RM is to provide method harmonization and eventual quality assurance and quality control (QA/QC) for measurements intended to capture gut microbiome and gut metabolome function. While this RM will be applicable across many scientific disciplines, it will be rationally designed and characterized to be especially well-suited to support those in the scientific community studying the impact of diet and nutrition on functional aspects of the host microbiome as well as the relationship between the host microbiome and human health. To identify which metabolites deserve priority in their value assignment, the three parties jointly conducted a workshop on September 12, 2019 at the NIST campus in Gaithersburg, Maryland. The objectives of this workshop were to identify metabolites for which evidence indicates relevance to health and disease and to decide on the appropriate course of action to develop fit-for-purpose RM(s). This document represents the consensus opinions of the workshop participants and co-authors of this manuscript. In addition, this paper describes the goals of the whole stool RM project, it offers a consensus approach for NIST’s development of the whole stool RM, and it addresses some of the strengths and limitations of going forward with this initiative given the current state of microbiome research.

Overall, the goals of this RM project are to (1) develop a human gut microbiome RM prepared from human stools, (2) promote the adoption of this RM by the scientific community to validate and benchmark current protocols, and (3) promote adoption of the RM as the “gold standard” for QC of metagenomic and metabolomic measurements of clinically relevant stool materials.

## Current knowledge of human fecal sample collection, preparation, and analysis

### Sample collection and storage

Due to the complexity of the human stool matrix, stool samples reflect more than the environment of the host gut. Stool samples are susceptible to environmental effects. Significant post-collection metabolite deterioration occurs due to exposure to aerobic conditions and to temperature changes, mainly caused by microbial fermentation at room temperature. In many of the reviewed studies, it has been indicated that stool samples were immediately stored at 4 °C or lower until processing (Gratton et al. 2016). For a metabolomics study design, the collection methodology should be kept as consistent as possible across all samples in the experimental classes to avoid bias.

Sample collection is a large source of variability across studies. A comparison between sample preparation conditions and their effects on stool metabolite detection is given in a recent review paper (Karu et al. 2018). The use of frozen stool samples in human studies is both more common and somewhat more practical. However, freeze-drying is also an option but may result in the loss of volatile compounds, many of which have been linked to physiological benefits and are thus of research interest. For fecal collections at home or in locations where immediate freezing is not possible, OMNIgene Gut Stool Microbiome Kits (Anderson et al. 2016) and others like it may be used. These kits contain reagents to stabilize and preserve samples at ambient temperatures, allowing for short-term storage and shipping without compromising sample and data integrity (Liang et al. 2020; Wang et al. 2018). Homogenizing and aliquoting samples prior to freezing can minimize unintended or unnecessary freeze–thaw cycles. As with other biofluids or tissue specimens in general, it is important to minimize handling time and to use uniform sample handling/storage procedures across the sample set. Reporting an averaged value for specific metabolite mass fractions or concentrations for samples collected over multiple days is also a common strategy for reducing the apparent variability.

Spot versus total fecal sampling is also an important consideration because it influences how data are interpreted. While concentrations (e.g., µmol/g digesta or feces) are usually the focus, the total metabolite pools (e.g., total µmol in colon; total µmol in body) are just as, or even more important. Exercise, dietary intake (e.g., fiber), obesity, and other environmental factors may impact intestinal transit time/defecation frequency, total volume of digesta/feces excreted, fecal moisture content, and microbial density. These variables may consequently affect metabolite production rates, digesta/fecal concentrations, and total exposure to the host (Falony et al. 2018). From a host energetics and health perspective, for example, the total SCFA produced and utilized by the body has much greater relevance than a fecal concentration (Bergman 1990; Dalile et al. 2019). Thus, calculating the total gastrointestinal SCFA pool from the SFCA concentration and the total volume of digesta/feces will provide greater insight than concentration alone. Likewise, the impact of bile acids on the body is likely not well described by fecal concentrations because the body’s pool includes the serum, liver, gallbladder (bile), gastrointestinal tract (duodenum, jejunum, ileum, cecum, and colon), and fecal components (Chiang and Ferrell 2020). Lastly, from a gastrointestinal disease perspective (e.g., colorectal cancer; inflammatory bowel diseases), the digesta/fecal concentrations of toxic compounds (e.g., phenols and indoles; hydrogen sulfide; heterocyclic amines; branched-chain fatty acids), transit time, and digesta/fecal volume may be used to estimate total exposure to the body (Ikeda et al. 1994; Le Gall et al. 2018). Given the number of factors involved and their influences on health, total fecal collections over a period of a few days, in addition to the fresh samples used for metabolite concentration measurement, are suggested so that total exposures may be estimated.

### Challenges in fecal sample preparation

As human stool samples contain an extraordinary variety of chemical classes, a true representation of the metabolic profile requires broad and consistent metabolite recovery during the extraction process. However, by maximizing metabolite recovery, the extraction process should not be destructive, nor should it alter the bacterial content of human stools (Gratton et al. 2016). The risk for this is reduced when samples are filtered, dried, or undergo sterilization by means of sonication (Saric et al. 2008). However, these procedures may increase the risk of metabolite degradation or loss, depending on the chemical or physical “aggressiveness” of the applied extraction method. The optimal extraction processes also depend on the study objectives. If the focus of a study is on intracellular bacterial metabolites and membrane lipids, a more vigorous homogenization may be required to lyse the bacterial cells. This may also be promoted via multiple freeze–thaw cycles (Shao et al. 2016, 2017). The use of a multi-step extraction procedure is another source of variation in metabolite recovery. It is recommended that the reproducibility of a multi-step recovery can be partly assessed (per chemical class) by the addition of isotopically labeled internal standards at the start of each sample extraction, along with appropriate sample homogenization to mix the standards into the matrix (Saric et al. 2008).

The quantity of starting material is another important factor that affects the reproducibility of the extraction recovery. Samples less than 150 mg prior to freeze-drying tend to suffer from relatively higher deviations in measured weight as well as chemical perturbations due to the introduction of external contaminants (Cesbron et al. 2017; Deda et al. 2015). It is generally recommended that blank extractions be added to the set of actual sample extractions to prevent misinterpretation of the collected NMR spectra (Marchesi et al. 2007).

The issue of water content in stool samples also influences the extraction recovery and metabolite concentrations reported. First, if appropriate care is not taken to achieve good extraction reproducibility, it is possible to have situations where the technical variance is greater than the biological variance, leading to incorrect conclusions. Second, water content of feces may vary considerably. Human feces commonly contain 60% to 80% water (by mass), which can decrease or increase beyond this range in cases of constipation and diarrhea, respectively (Nishimuta et al. 2006). Given the high water content of feces and the wide range by which it may fluctuate within and among study participants, representing fecal metabolite data on a wet or dry basis is not a minor consideration.

### Analysis of human stool samples

The success of a stool metabolomics study depends on having an appropriate analytical approach and access to suitable analytical platforms in addition to adequate sample collection and preparation protocols. As highlighted in a recent review, there are no best practices for metabolomic analyses of human stool material (Karu et al. 2018). The lack of consensus best practices can lead to tremendous variability in reported results. In addition, for many metabolomic studies, the metabolite sensitivity and coverage can vary tremendously between different types of instruments or different types of platforms. It is highly recommended that more than one analytical platform is used because (1) different platforms often have complementary sensitivity to different classes of metabolites (gas chromatography [GC]-mass spectrometry [MS] for volatiles and organic acids, nuclear magnetic resonance [NMR] for very polar compounds, and liquid chromatography [LC]-MS for more hydrophobic molecules); (2) using more than one platform can greatly increase the breadth of metabolite coverage; and (3) detection and/or quantification of a metabolite on one or more platform helps with confirmation and instrument calibration.

LC–MS methods for fecal metabolomics provide relatively wide metabolite coverage compared to that of NMR and GC–MS assays. This can be further extended by the application of careful LC separations coupled with multiple ionization technologies and polarities (Nordstrom et al. 2008). High-throughput approaches in LC–MS are advancing quickly owing to the development of ultra-performance LC systems and polarity switching, which is now widely available on many high-resolution MS instruments. These advantages have already contributed to the dramatic increase in utilization of LC–MS in stool analysis or fecal metabolomics over the past 3 years to 4 years (Karu et al. 2018).

## NIST’s intention to produce a metagenomic and metabolomic reference material

NIST has a unique global role as a National Metrology Institute for the United States but also as a non-regulatory agency in the U.S. Department of Commerce with a mission to promote U.S. innovation and industrial competitiveness by advancing measurement science, standards, and technology. While serving as the national reference laboratory for measurements in the chemical, biological, and material sciences, NIST has developed RMs that are designed to validate analytical methods used in the determination of nutritional and health status markers, contaminant exposure components, and clinically relevant metabolites in human serum, plasma, and urine (National Institute of Standards and Technology 2020a, b; Rasberry 2003). One of the more notable is Standard Reference Material 1950 – Metabolites in Frozen Human Plasma, which has been value assigned (i.e., certified and non-certified mass fraction content) for nearly 100 electrolytes, vitamins, hormones, fatty acids, and amino acids, among other chemical species (Simon-Manso et al. 2013). Currently, suites of pooled human plasma and urine RMs for untargeted metabolome analysis are under development. These material suites will be characterized with respect to both the chemical annotation/identification of the predominant metabolites and their respective fold changes/percent differences for purposes of underpinning differential metabolomics and lipidomics studies.

NIST is presently developing a candidate Human Gut Microbiome (Whole Stool) RM for measurement harmonization and QA/QC for stool-based metagenomics, metabolomics, and clinical measurements. The most common measurements currently being used in gut microbiome research and sample testing laboratories are next generation sequencing (NGS)-based metagenomics and MS-based metabolomics. However, in either case, no fit-for-purpose RMs exist that enable researchers to compare results generated across different laboratories and to assess the impact of the multitude of methodological variables that exist in either measurement platform. To begin to understand the biologically relevant properties of the human gut microbiome, the community needs such RMs for confident identification of relevant biomarkers that may serve as health or disease indicators while supporting the validation of analytical measurements (mass fraction or mass concentration) for clinically relevant metabolites and/or nutritional assessment metabolic markers.

This candidate Human Whole Stool RM will comprise two types of human whole stool materials—one from healthy donors who are omnivores and one from healthy vegan and vegetarian donors. The homogeneity and stability, with respect to the metabolomic and metagenomic profiles of their complex microbial communities, of both materials will be thoroughly assessed. Similar to the NIST metabolomics plasma and urine RMs (in the development pipeline), the predominant metabolites will be annotated/identified before the RM is made publicly available. The metabolites annotated or identified will primarily be those for which current evidence supports a link to physiological effects or better health outcomes, and those which are consistently present in human stool at high levels. Additionally, DNA will be quantified and metagenomic profiles will be evaluated via NGS. Certification of any preliminary value assignments may not be necessary for the initial harmonization of metabolomics-based whole stool measurements. However, NIST can evolve its RMs toward Certified Reference Materials (CRMs) when deemed necessary by stakeholders. Thus, if a targeted metabolic biomarker is identified as a critical measure or indicator for stool-based measurements, options are available to “upgrade” the Human Whole Stool RM to include certified values. This generally involves conducting further measurements exclusively at NIST using reference measurement procedures or additional higher-order measurement procedures that are often metrologically traceable to the SI.

NIST has already begun working with TBC to evaluate their human whole stool preparation and preservation methodologies to determine how best to stabilize and store candidate RMs for both metagenomic and metabolomic measurements over an extended period. Additionally, NIST is working with ILSI North America to determine metabolites of interest (i.e., linked to health outcomes) that should be annotated/identified in the RM even if they are not the most dominant biochemical species present.

## NIST pilot study on stool stability and homogeneity

**Fig. 1:**
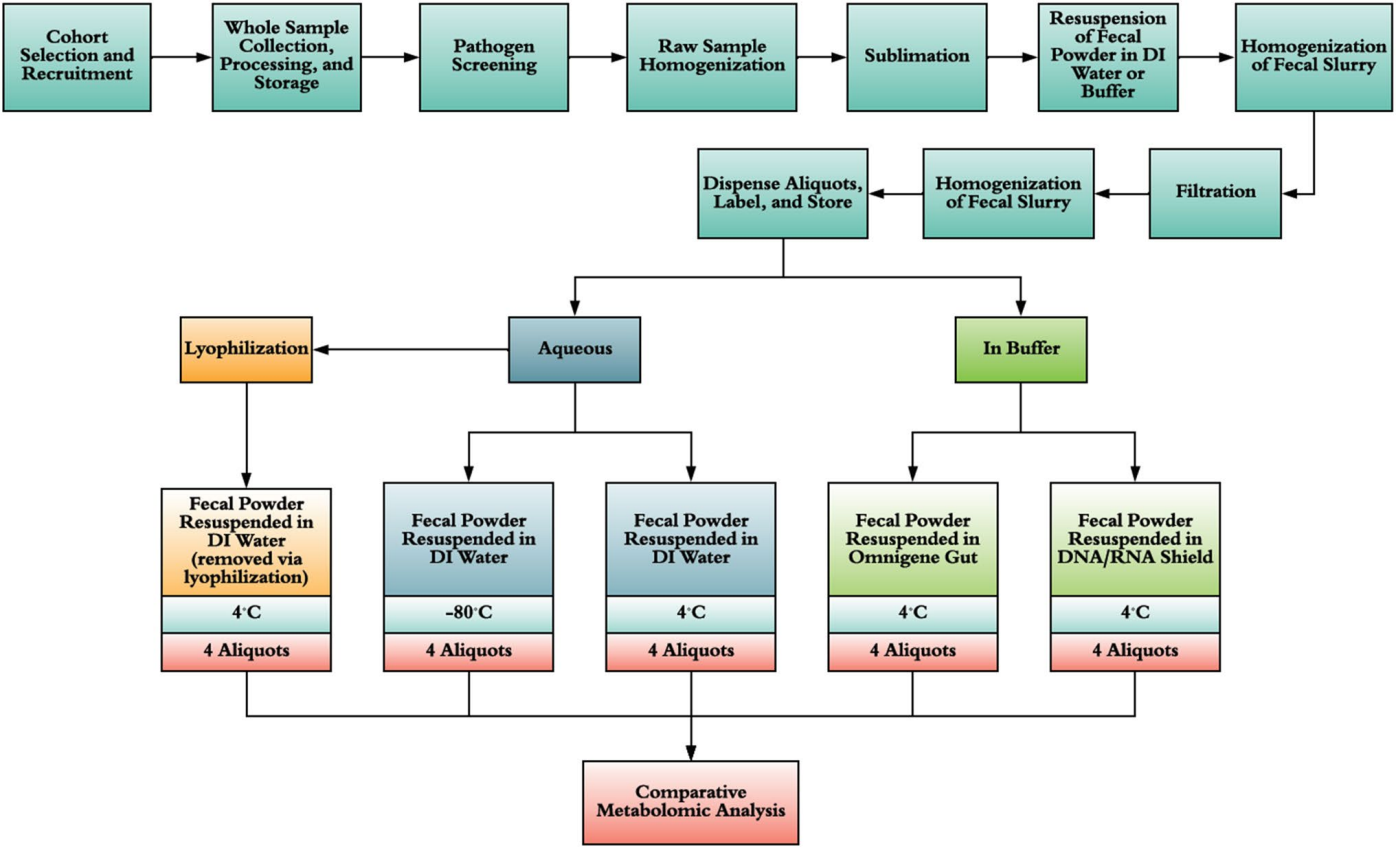
Manufacturing flow of the TruMatrix™ (The BioCollective, LLC) Microbiome Reference Material

As a first step to development of the Human Whole Stool RM, the candidate materials need to be stable and homogeneous for specified targeted measurands. We performed a pilot study to investigate stool sample storage conditions that could potentially stabilize the targeted metabolites present in the material prepared by TBC using its proprietary technologies for processing, stabilization, and storage. The production of candidate stool RMs will be based largely on the manufacturing process illustrated in Fig. 1. A similar process was used to produce the material employed in this pilot study.

**Fig. 2:**
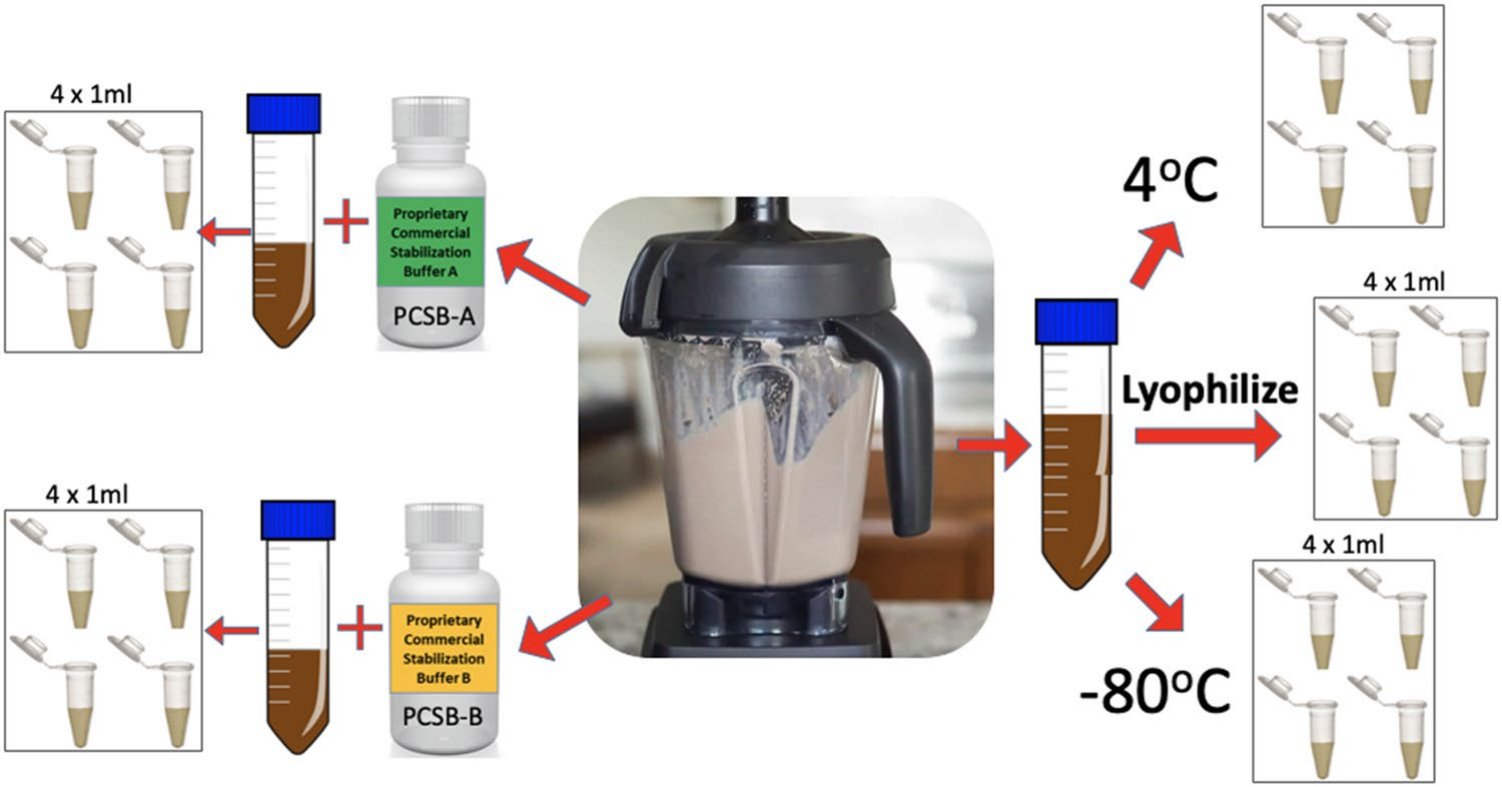
Manufacturing flow for the five different stabilization methods used for the homogenized stool sample

Metabolites were extracted from stool samples that were either homogenized in water and stored at − 80 °C or 4 °C, lyophilized and stored at 4 °C, or homogenized in a preservation buffer (two buffers were used, defined as 1 and 2) and stored at 4 °C. In total, five different conditions were analyzed as shown in Fig. 2. The ideal preservation technique would be amenable to various measurement platforms (fit-for-purpose). For the purposes of this pilot study, microbial taxa and metabolite patterns across multiple aliquots from the same storage condition were evaluated and used to estimate homogeneity within that storage condition. Extensive homogeneity studies will be performed on the candidate RM in the future, once preservation methods have been optimized. The stool samples were stored for 4 months under the above conditions prior to analysis. This pilot study did not include any analyses of the freshly prepared material (t = 0 timepoint), therefore stability over time could not be assessed. Only the homogeneity and the impact of the various storage conditions was assessed.

Samples were sent to NIST from TBC and analyzed via NMR-based metabolomics, MS-based metabolomics, and 16S rRNA amplicon DNA sequencing. These three analytical approaches provide a picture of the taxonomic and metabolic profiles of the microbial community (Butlen and Jard 1972; Wishart 2019b). MS and NMR are complementary approaches in the detection of small molecules (< 1.5 kDa) and widely used analytical platforms for untargeted metabolome characterization. In terms of RM development, MS-based metabolomics covers a large range of metabolites due to its analytical sensitivity, thus it constitutes an ideal technique for quantifying low-level biomarkers. MS-based analyses often require a separation on the front end of the workflow. GC is ideal for the detection of volatile and semi-volatile compounds, which are highly abundant in whole stool samples. LC covers a wide range of compounds and is regarded as the gold standard separation technique for metabolomics. Both GC–MS and LC–MS analyses are prone to reproducibility challenges due to batch effects and the innate instability of MS, which can be mitigated through careful sample preparation and the use of labeled internal standards. Utilizing NMR for gut microbiome metabolomic studies is advantageous as this technique offers high reproducibility and absolute quantification. Nearly all protons have an equivalent response across molecules. Moreover, it offers simplicity in sample preparation. Metabolite profiles obtained by NMR are virtually independent of the operator and instrument parameters, which provides a high degree of reliability to the results. Additionally, since samples do not interact directly with the instrument, NMR analysis is free from batch effects such as carryover. However, NMR does not provide a global analysis due to low sensitivity, which is typically in the micromolar range as opposed to the nanomolar range provided by MS. NMR is an ideal analysis for monitoring the stability and homogeneity of the metabolites present in a fecal RM as well as absolute quantification of some metabolites.

To keep within the scope of this manuscript, which is to summarize the September 2019 workshop, we focus here on a global discussion of the sample analysis. The five preservation methods presented unique challenges for the characterization of the samples. In the NMR analysis, pH differences among the samples led to peak shifting of acidic metabolites such as acetic acid. Additionally, the use of ethanol in the proprietary commercial preservation solutions significantly hindered spectral analysis by introducing strong peaks in multiple regions of the spectrum that were much larger than neighboring metabolite peaks. In LC–MS analysis, one of the proprietary buffers contained extremely high levels of the DNA stabilizer, N-dodecanoyl-N-methylglycine, which led to a large interference in the analysis and instrument contamination. Overall, the results of all analyses were consistent with each other in that within a preservation technique, the metabolite profiles appear to be homogeneous. However, different preservation techniques introduce varying metabolite biases. 16S rRNA amplicon gene sequencing indicated changes in the relative abundance of the microbial taxa that presumably occurred during storage under different conditions. The microbial community profile of the samples homogenized in water and stored at 4 °C was most different from all other preservation and storage conditions, likely indicating relative abundance shifts in the microbial population due to potential growth during storage. In fact, many bacterial species can sustain growth at 4 °C, particularly given the abundance of nutrients in fecal material. The results of the combined metagenomic and metabolomic pilot study suggest that lyophilized samples as well as samples stored at – 80 °C are the most suitable for RM development.

## Criteria for reference material metabolite selection

One of the key objectives of the September 2019 meeting was to develop a consensus for the stool RM metabolite selection. Metabolites for further vetting were initially generated using three approaches: (i) expert consensus on metabolites of health relevance (Table 1); (ii) microbially produced compounds as catalogued in the Fecal Metabolome Database (www.fecalmetabolome.ca) (Table 2); and (iii) a PubMed search with chemical synonyms (Table 3). Tables in this paper were generated through an analysis of a number of online databases as of September 2019, such as the HMDB (Wishart et al. 2018; www.hmdb.ca), the fecal metabolome database (www.fecalmetabolome.ca), as well as through literature reviews conducted by the authors and feedback provided by workshop participants.

**Table 1:** Potential list of candidate fecal metabolites of health relevance

*Candidate metabolites*
Bilirubin
Butyric acid
Enterolactone (bacterial metabolite of plant lignans) and/or its glucuronide and sulfate conjugates
Odd chain fatty acid such as 15:0 or 17:0
Lithocholic acid (secondary bile acid) and/or glycine and taurine conjugates (or other secondary bile acid)
Hippuric acid
Indole
Isoflavone derivatives (for complex ring structures)
p-Cresol
Putrescine
Phenyl lactic and p-hydroxyphenyl lactic acid
Trimethylamine oxide (bacterial trimethylamine oxidized to TMAO in liver)

*TMAO* trimethylamine N-oxide

This list was generated by the planning committee and brought to the workshop for participant reaction

**Table 2:** List of candidate microbially produced compounds from the Fecal Metabolome Database (www.fecalmetabolome.ca)

Compound name^a^	InChi key
SCFAs	
Propionic acid	XBDQKXXYIPTUBI-UHFFFAOYSA-N 1
Acetic acid	QTBSBXVTEAMEQO-UHFFFAOYSA-N 1
Formic acid	BDAGIHXWWSANSR-UHFFFAOYSA-N 1
Isobutyric acid	KQNPFQTWMSNSAP-UHFFFAOYSA-N 1
Valeric acid	NQPDZGIKBAWPEJ-UHFFFAOYSA-N 1
Isovaleric acid	GWYFCOCPABKNJV-UHFFFAOYSA-N 1
(S)-3,4-Dihydroxybutyric acid	DZAIOXUZHHTJKN-UHFFFAOYSA-N 1
1,2,3-Propanetricarboxylic acid	KQTIIICEAUMSDG-UHFFFAOYSA-N 1
1,2,3-Trihydroxybenzene	WQGWDDDVZFFDIG-UHFFFAOYSA-N 1
1-Butanol	LRHPLDYGYMQRHN-UHFFFAOYSA-N 1
1-Deoxy-d-xylulose 5-phosphate	AJPADPZSRRUGHI-RFZPGFLSSA-N 1
1H-Indole-3-carboxaldehyde	OLNJUISKUQQNIM-UHFFFAOYSA-N
2,3-Butanediol	OWBTYPJTUOEWEK-UHFFFAOYSA-N 1
2,5-Furandicarboxylic acid	CHTHALBTIRVDBM-UHFFFAOYSA-N 1
2-Hydroxycaproic acid	NYHNVHGFPZAZGA-UHFFFAOYSA-N 1
2-Hydroxyglutarate	HWXBTNAVRSUOJR-UHFFFAOYSA-N 1
2-Isopropylmalic acid	BITYXLXUCSKTJS-ZETCQYMHSA-N 1
2-Keto-3-deoxy-6-phosphogluconic acid	OVPRPPOVAXRCED-WVZVXSGGSA-N 1
2-Oxo-3-phenylpropanoic acid (Mixture oxo and keto)	BTNMPGBKDVTSJY-UHFFFAOYSA-N 0
2-oxoglutarate(2-)	KPGXRSRHYNQIFN-UHFFFAOYSA-L 0
2-Phenylpropionic acid	YPGCWEMNNLXISK-UHFFFAOYSA-N
3-(3-Hydroxyphenyl)-3-hydroxypropanoic acid	KHTAGVZHYUZYMF-UHFFFAOYSA-N 1
3-(3-Hydroxyphenyl)propanoic acid	QVWAEZJXDYOKEH-UHFFFAOYSA-N 1
3-(Methylthio)-1-propene	NVLPQIPTCCLBEU-UHFFFAOYSA-N 1
3,4-Dihydroxybenzeneacetic acid	CFFZDZCDUFSOFZ-UHFFFAOYSA-N 1
3,4-Dihydroxyhydrocinnamic acid	DZAUWHJDUNRCTF-UHFFFAOYSA-N 1
3-Hydroxybenzoic acid	IJFXRHURBJZNAO-UHFFFAOYSA-N 1
3-Hydroxybutyric acid	WHBMMWSBFZVSSR-GSVOUGTGSA-N 1
3-Hydroxyhippuric acid	XDOFWFNMYJRHEW-UHFFFAOYSA-N 1
3-Hydroxyphenylacetic acid	FVMDYYGIDFPZAX-UHFFFAOYSA-N 1
3-Indolebutyric acid	JTEDVYBZBROSJT-UHFFFAOYSA-N 1
3-Methylindole	ZFRKQXVRDFCRJG-UHFFFAOYSA-N 1
3-O-Methylgallic acid	KWCCUYSXAYTNKA-UHFFFAOYSA-N 0
4-Hydroxybenzoic acid	FJKROLUGYXJWQN-UHFFFAOYSA-N 1
4-Hydroxybutyric acid	SJZRECIVHVDYJC-UHFFFAOYSA-N 1
4-Hydroxycyclohexylcarboxylic acid	HCFRWBBJISAZNK-UHFFFAOYSA-N 1
4-Hydroxyvaleric acid	FMHKPLXYWVCLME-UHFFFAOYSA-N 0
4-O-Methylgallic acid	UBXDWYFLYYJQFR-UHFFFAOYSA-N 1
5-(3′,4′-Dihydroxyphenyl)-gamma-valerolactone	ZNXXWTPQHVLMQT-MRVPVSSYSA-N 1
5-Amino-6-(5′-phosphoribitylamino)uracil	RQRINYISXYAZKL-RPDRRWSUSA-N 1
5beta-Coprostanol	QYIXCDOBOSTCEI-NWKZBHTNSA-N 1
5-Hydroxyhexanoic acid	YDCRNMJQROAWFT-UHFFFAOYSA-N 1
5-Hydroxyindoleacetic acid	DUUGKQCEGZLZNO-UHFFFAOYSA-N 1
5-Hydroxypentanoic acid	PHOJOSOUIAQEDH-UHFFFAOYSA-N 1
5-Keto-d-gluconate	IZSRJDGCGRAUAR-UHFFFAOYSA-N 1
6,7-Dimethyl-8-(1-d-ribityl)lumazine	SXDXRJZUAJBNFL-XKSSXDPKSA-N 1
Acetaldehyde	IKHGUXGNUITLKF-UHFFFAOYSA-N 1
Acetone	CSCPPACGZOOCGX-UHFFFAOYSA-N 1
Acetylcholine	OIPILFWXSMYKGL-UHFFFAOYSA-N 1
ADP-glucose	WFPZSXYXPSUOPY-ROYWQJLOSA-N 1
Allantoin	POJWUDADGALRAB-UHFFFAOYSA-N 1
Ascorbic acid	CIWBSHSKHKDKBQ-JLAZNSOCSA-N 1
Benzoic acid	WPYMKLBDIGXBTP-UHFFFAOYSA-N 1
Benzoyl-CoA	VEVJTUNLALKRNO-TYHXJLICSA-N 1
Cadaverine	VHRGRCVQAFMJIZ-UHFFFAOYSA-N 1
Caffeic acid	QAIPRVGONGVQAS-DUXPYHPUSA-N 1
cis,cis-Muconic acid	TXXHDPDFNKHHGW-CCAGOZQPSA-N 1
Citramalic acid	XFTRTWQBIOMVPK-UHFFFAOYSA-N 1
d-Alanine	QNAYBMKLOCPYGJ-UWTATZPHSA-N 1
d-Arabinose	SRBFZHDQGSBBOR-SQOUGZDYSA-N 1
d-Arabitol	HEBKCHPVOIAQTA-QWWZWVQMSA-N 1
Desaminotyrosine	NMHMNPHRMNGLLB-UHFFFAOYSA-N 1
d-Glutamic acid	WHUUTDBJXJRKMK-GSVOUGTGSA-N 1
Diaminopimelic acid	GMKMEZVLHJARHF-WHFBIAKZSA-N 1
Dimethyl sulfone	HHVIBTZHLRERCL-UHFFFAOYSA-N 1
Dimethyl trisulfide	YWHLKYXPLRWGSE-UHFFFAOYSA-N 1
Dimethylamine	ROSDSFDQCJNGOL-UHFFFAOYSA-N 1
Dimethylsulfide	QMMFVYPAHWMCMS-UHFFFAOYSA-N 1
d-Lactic acid	JVTAAEKCZFNVCJ-UWTATZPHSA-N 1
Dopamine	VYFYYTLLBUKUHU-UHFFFAOYSA-N 1
d-Ribose	HMFHBZSHGGEWLO-SOOFDHNKSA-N 1
d-Ribulose	LQXVFWRQNMEDEE-UOWFLXDJSA-N 1
Ethanol	LFQSCWFLJHTTHZ-UHFFFAOYSA-N 1
Etiocholanedione	RAJWOBJTTGJROA-QJISAEMRSA-N 1
Folic acid	OVBPIULPVIDEAO-LBPRGKRZSA-N 1
Galactan	PTHCMJGKKRQCBF-ICIGWGHZSA-N 1
Gallic acid	LNTHITQWFMADLM-UHFFFAOYSA-N 1
gamma-Aminobutyric acid	BTCSSZJGUNDROE-UHFFFAOYSA-N 1
Gentisic acid	WXTMDXOMEHJXQO-UHFFFAOYSA-N 1
Glutaric acid	JFCQEDHGNNZCLN-UHFFFAOYSA-N 1
Glyceric acid	RBNPOMFGQQGHHO-UWTATZPHSA-N 1
Glycocholic acid	RFDAIACWWDREDC-FRVQLJSFSA-N 1
Glycolic acid	AEMRFAOFKBGASW-UHFFFAOYSA-N 1
Homogentisic acid	IGMNYECMUMZDDF-UHFFFAOYSA-N 1
Homovanillic acid	QRMZSPFSDQBLIX-UHFFFAOYSA-N 1
Hydrocinnamic acid	XMIIGOLPHOKFCH-UHFFFAOYSA-N 1
Hydroxyisocaproic acid	LVRFTAZAXQPQHI-YFKPBYRVSA-N 1
Hydroxyphenyllactic acid	JVGVDSSUAVXRDY-UHFFFAOYSA-N 1
Hydroxypropionic acid	ALRHLSYJTWAHJZ-UHFFFAOYSA-N 1
Hydroxytyrosol	JUUBCHWRXWPFFH-UHFFFAOYSA-N 1
Indole	SIKJAQJRHWYJAI-UHFFFAOYSA-N 1
Indole-3-methyl acetate	KTHADMDGDNYQRX-UHFFFAOYSA-N 1
Indole-3-propionic acid	GOLXRNDWAUTYKT-UHFFFAOYSA-N 1
Indoleacetic acid	SEOVTRFCIGRIMH-UHFFFAOYSA-N 1
Indoleacrylic acid	PLVPPLCLBIEYEA-AATRIKPKSA-N 1
Indolepyruvate	RSTKLPZEZYGQPY-UHFFFAOYSA-N 1
Indoxyl sulfate	BXFFHSIDQOFMLE-UHFFFAOYSA-N 1
Isobutanol	ZXEKIIBDNHEJCQ-UHFFFAOYSA-N 1
Isopropyl alcohol	KFZMGEQAYNKOFK-UHFFFAOYSA-N 1
L-Gulose	WQZGKKKJIJFFOK-QRXFDPRISA-N 1
Lipid A	GZQKNULLWNGMCW-PWQABINMSA-N 1
L-Lactic acid	JVTAAEKCZFNVCJ-REOHCLBHSA-N 1
L-Rhamnulose	QZNPNKJXABGCRC-OTWZMJIISA-N 1
l-Sorbose	LKDRXBCSQODPBY-BGPJRJDNSA-N 1
Mannitol	FBPFZTCFMRRESA-KVTDHHQDSA-N 1
Mannitol 1-phosphate	GACTWZZMVMUKNG-KVTDHHQDSA-N 1
m-Cresol	RLSSMJSEOOYNOY-UHFFFAOYSA-N 1
Melibiose	DLRVVLDZNNYCBX-CQHUIXDMSA-N 1
Methylisocitric acid	HHKPKXCSHMJWCF-UHFFFAOYSA-N 1
Monodehydroascorbate	PIECNOFUNCLXPG-SAGVERJBSA-N 0
N-Acetylmannosamine	OVRNDRQMDRJTHS-OZRXBMAMSA-N 1
N-Acetylputrescine	KLZGKIDSEJWEDW-UHFFFAOYSA-N 1
N-benzoylglycinate	QIAFMBKCNZACKA-UHFFFAOYSA-N 0
Nonadecanoic acid	ISYWECDDZWTKFF-UHFFFAOYSA-N 1
Norepinephrine	SFLSHLFXELFNJZ-QMMMGPOBSA-N 1
o-Cresol	QWVGKYWNOKOFNN-UHFFFAOYSA-N 1
Oxalic acid	MUBZPKHOEPUJKR-UHFFFAOYSA-N 1
Oxoglutaric acid	KPGXRSRHYNQIFN-UHFFFAOYSA-N 1
p-Aminobenzoic acid	ALYNCZNDIQEVRV-UHFFFAOYSA-N 1
p-Cresol sulfate	WGNAKZGUSRVWRH-UHFFFAOYSA-N 1
Phenol	ISWSIDIOOBJBQZ-UHFFFAOYSA-N 1
Phenylacetylglutamine	JFLIEFSWGNOPJJ-JTQLQIEISA-N 1
Phenylethylamine	BHHGXPLMPWCGHP-UHFFFAOYSA-N 1
Phenyllactic acid	VOXXWSYKYCBWHO-UHFFFAOYSA-N 1
Phloretin	VGEREEWJJVICBM-UHFFFAOYSA-N 1
p-Hydroxyphenylacetic acid	XQXPVVBIMDBYFF-UHFFFAOYSA-N 1
Propyl alcohol	BDERNNFJNOPAEC-UHFFFAOYSA-N 1
Protocatechuic acid	YQUVCSBJEUQKSH-UHFFFAOYSA-N 1
Putrescine	KIDHWZJUCRJVML-UHFFFAOYSA-N 1
Pyrocatechol	YCIMNLLNPGFGHC-UHFFFAOYSA-N 1
Pyrrolidine	RWRDLPDLKQPQOW-UHFFFAOYSA-N 1
Pyruvic acid	LCTONWCANYUPML-UHFFFAOYSA-N 1
Rhamnose	SHZGCJCMOBCMKK-JFNONXLTSA-N 1
Salicylic acid	YGSDEFSMJLZEOE-UHFFFAOYSA-N 1
Serotonin	QZAYGJVTTNCVMB-UHFFFAOYSA-N 1
Sorbose 1-phosphate	ZKLLSNQJRLJIGT-OTWZMJIISA-N 1
Stercobilin	DEEUSUJLZQQESV-BQUSTMGCSA-N 1
Succinic acid	KDYFGRWQOYBRFD-UHFFFAOYSA-N 1
Sulfoglycolithocholate(2-)	FHXBAFXQVZOILS-UHFFFAOYSA-N 1
Sumiki’s acid	PCSKKIUURRTAEM-UHFFFAOYSA-N
Syringic acid	JMSVCTWVEWCHDZ-UHFFFAOYSA-N 1
Tartaric acid	FEWJPZIEWOKRBE-JCYAYHJZSA-N 1
Taurodeoxycholic acid	AWDRATDZQPNJFN-VAYUFCLWSA-N 1
trans-Cinnamic acid	WBYWAXJHAXSJNI-VOTSOKGWSA-N 1
trans-Ferulic acid	KSEBMYQBYZTDHS-HWKANZROSA-N
Trehalose	HDTRYLNUVZCQOY-LIZSDCNHSA-N 1
Trehalose 6-phosphate	LABSPYBHMPDTEL-LIZSDCNHSA-N 1
Tryptamine	APJYDQYYACXCRM-UHFFFAOYSA-N 1
Tryptophanol	MBBOMCVGYCRMEA-UHFFFAOYSA-N
Tuberculostearic acid	BEOUGZFCUMNGOU-UHFFFAOYSA-N 1
Tyraminium	DZGWFCGJZKJUFP-UHFFFAOYSA-O 0
Tyrosol	YCCILVSKPBXVIP-UHFFFAOYSA-N 1
Vanillic acid	WKOLLVMJNQIZCI-UHFFFAOYSA-N 1
Zeaxanthin	JKQXZKUSFCKOGQ-QAYBQHTQSA-N 1

^a^Compounds are listed in alphabetical order from top to bottom, left to right. The international chemical identifier (InChi) keys can be found at www.hmdb.ca

Four different routes that can be used to determine if a metabolite is microbially or host produced: (1) literature review, (2) published pathways, (3) genomic reconstruction evidence, and (4) evidence from experimental work for robogut (a bioreactor that allows researchers to study the colon) and SHIME (The Simulator of the Human Intestinal Microbial Ecosystem) studies

**Table 3:** Typical microbe-source compounds: based on PubMed search with chemical synonyms

Metabolite	Reference count
Ethanol	3233
Phenol	2444
Folic acid	2253
l-Lactic acid	2020
Acetic acid	2006
d Lactic acid	1512
Butyric acid	1426
Benzoic acid	1172
Indole	1080
Propionic acid	911
Formic acid	699
Bilirubin	586
d-Alanine	569
d-Glutamic acid	547
Acetaldehyde	531
2-Hydroxyglutarate	485
Galactan	437
Serotonin	407
Pyrocatechol	385
Lithocholic acid	319
Pentadecanoic acid	278
Ascorbic acid	256
Succinic acid	238
Mannitol	225
Pyruvic acid	221
Oxalic acid	190
Propyl alcohol	184
Tryptamine	177
Valeric acid	177
Phenylethylamine	167
Dopamine	134
Acetone	131
Acetylcholine	128
p-Cresol	126
Hippuric acid	121
Norepinephrine	120
Gallic acid	117
Isobutyric acid	108
Rhamnose	102

Search results through August 12, 2019 are included. Only metabolites with 100 or more indicated references were included. Searches were conducted using PubMed with chemical synonyms and were based on abstracts only

The following general principles were proposed as criteria for selecting metabolites to be eventually characterized in the RM: The metabolites should (1) have health or disease relevance, (2) be identifiable as part of a known biological pathway, (3) be common in a diversity of fecal samples, (4) have demonstrated robustness in the analytical method used for measurement, (5) be considered in light of the variety of analytical platforms that may be used and research foci, (6) include specific analytes that are challenging to measure (considering retention time, increased volatility, trace concentration levels, etc.), and finally, (7) include analytes with a range of chemical stability that are known to change over time frames relevant to RM production.

A modified list of potential candidate metabolites was generated through discussion groups and report-back during the September 19 event (Table 4) from a careful review of Tables 1, 2 and 3 and considering the criteria outlined above. This list and the accompanying criteria are being provided to NIST as a guide as they engage in the whole stool RM development process. At present, knowledge is limited with respect to how sample collection affects the presence and measurement of these metabolites, how well metabolite profiles in fecal samples represent digesta in the gastrointestinal tract, and which metabolites are linked to measurable physiological effects or health benefits. It is recognized that the metabolites characterized in the stool RM can, and will, evolve in parallel with progressive developments in stool metabolomic approaches as well as advances in nutrition and gut microbiome science, as linked to health and disease. Future opportunities include understanding the full spectrum of health relevance for various metabolite classes, and consideration of individuals consuming various diets (e.g., high fiber, strict vegan, high animal protein/fat) and with various disease states/conditions (e.g., irritable bowel disease/syndrome [active], obese vs. lean). Prior to the creation of a stool RM, a typical requirement is knowledge of the dynamic range of metabolites in a complex matrix.

**Table 4:** Modified list of potential metabolites for value assignment in a NIST Reference Material

Candidate metabolite	Alignment with proposed selection criteria^a^	Example references (alignment if specific)
SCFAs (butyric acid, propionic acid, acetic acid, formic acid)	1, 2, 3, 4, 6, 7, 8	Alexander et al. (2019) and Zhang et al. (2019) (4)
Succinic acid	1, 2, 3, 6	Fernández-Veledo and Vendrell (2019) and Xu and Wang (2016)
Lipids (e.g. linoleic acid; sphingomyelins [initially select based on abundance])	1, 2, 3, 6	Wang et al. (2016)
Deoxycholic acid	1, 2	Hullar and Fu (2014) (1) and Jewison et al. (2014) (2)
Enterohepatic metabolites (bacterial metabolites that are modified in the liver [TMAO])	1, 3, 6	Karu et al. (2018) (3) and Xu et al. (2015) (1, 6)
Tryptamine	1, 2, 6	Jewison et al. (2014) (2) and Williams et al. (2014) (1, 2)
Gallic acid	1, 6	Li et al. (2019b)
Tryptophan, serotonin (neurotransmitters)	1, 3, 2, 6	Karu et al. (2018) (3 for tryptophan), Jewison et al. (2014) (2) and Strandwitz (2018) (1)
Enterolactone (bacterial metabolite of plant lignans) and/or its glucuronide and sulfate conjugates	1	Halldin et al. (2019)
GABA	1, 3, 2, 6	Karu et al. (2018) (3), Strandwitz (2018) (1) and Jewison et al. (2014) (2)
Branched-chain fatty acids (isovaleric acid; isobutyric acid)	1, 2, 3	Abdallah et al. (2020) and Canfora et al. (2019)
Salicylic acid	1	Damman (2013)
Phenols and indoles (phenol, indole, p-cresol)	1, 3	Abdallah et al. (2020)
LPS with o-antigen (byproduct of Gram-negative bacteria)	1	Reyes et al. (2012)
Trans-ferulic acid	1	Xu and Wang (2016) and Agulló et al. (2020)
Mannitol	1	Xu and Wang (2016)
4-Pyridoxic acid	1	Obeid et al. (2019) (1)
Carnosine and trans-ferulic acid	1, 2, 6	Jewison et al. (2014) (2 for carnosine) and Schon et al. (2019) (1 for carnosine)
Theophylline	1	Cyr et al. (2008)

*GABA* gamma-aminobutyric acid, *LPS* lipopolysaccharide, *SCFA* short-chain fatty acid, *TMAO* trimethylamine N-oxide

This list is neither exhaustive nor definitive, but rather includes some options discussed by workshop participants

^a^Proposed criteria noted include that the metabolite: 1, is of health or disease relevance; 2, is identifiable as part of a known biological pathway; 3, is common in a diversity of fecal samples; 4, demonstrates confidence in the analytical method used for measurement; 5, is considered in light of the variety of analytical platforms that may be used and research foci; 6, is selected considering both technical and biological aspects; 7, is challenging to capture; 8, is an analyte of substantial stability or is known to change over time

## Conclusions and next steps for creating and evolving the material

Given the rapidly evolving nature of gut microbiome science and the current state of knowledge, an RM (as opposed to a CRM) measured for multiple metabolites is appropriate at this stage. As the science evolves, the RM can evolve to match the needs of the research community. Ultimately, the stool RM may exist in sequential versions. Beneficial to this evolution will be a clear line of communication between NIST and the stakeholder community to ensure alignment with current scientific understanding and community needs.

This document represents the consensus opinions of participants and co-authors of this manuscript, as captured in a September 12, 2019 workshop to explore creation of a human whole stool RM. ILSI North America, NIST, and TBC have engaged in Phase II of this effort which consists of a stability study of human stool material prepared from omnivore and vegan cohorts, each treated by freezing at − 80 °C or lyophilized. NIST will perform the metabolomics stability assessment on cohort aliquots prepared by TBC, including the following: (1) identifying “conserved” metabolites that can be used to assess homogeneity and stability, (2) assessing the effects of freeze–thaw cycles, (3) assessing microheterogeneity with respect to subsampling size, (4) assessing the effects of different extraction methods and solvents, (5) evaluating the presence of intracellular vs. extracellular metabolites, and (6) assessing the reproducibility across 5 to 10 laboratories through an interlaboratory study. With respect to metagenomic measurements, NIST will also assess the utility of spike-in organisms that can serve as pseudo-ground truth and provide an understanding of the analytical sensitivity and specificity of metagenomic measurements.

While there may be questions about whether this is the “right time” to develop a RM, it is important to remember that in much the same way that the metagenomics community has strived to develop and adopt standards, the functional microbiome and microbial metabolomic communities must also begin to develop and adopt standards for metabolomic measurements. In doing so, these measurements can be employed to make informed, clinically relevant, actionable decisions regarding the state of an individual’s gut microbiome, including disease predispositions and possible therapeutic or dietary intervention strategies aimed to benefit health.

## Data Availability

Not applicable.

## Abbreviations

CRM: Certified Reference Material
GC: Gas chromatography
ILSI North America: North American Branch of the International Life Sciences Institute
LC: Liquid chromatography
MS: Mass spectrometry
NGS: Next generation sequencing
NIST: National Institute of Standards and Technology
NMR: Nuclear magnetic resonance
PCA: Principal component analysis
QA/QC: Quality assurance and quality control
RM: Reference material
SCFA: Short-chain fatty acid
TBC: The BioCollective
